# Anesthetic Practices for Lower Segment Cesarean Section in the Sultanate of Oman: A National Survey

**DOI:** 10.7759/cureus.61204

**Published:** 2024-05-27

**Authors:** Abhijit Nair, Ramlaa Al Qasaab

**Affiliations:** 1 Anesthesiology, Ibra Hospital, Ibra, OMN; 2 Anesthesia and Critical Care, The Royal Hospital, Muscat, OMN

**Keywords:** anesthesia management, cesarean section (cs), survey research, perioperative anesthesia, obstetric anesthesia

## Abstract

Background

Over the years, obstetric anesthesia has evolved into a comprehensive sub-specialty. Several countries have their guidelines and recommendations for obstetric anesthesia. This survey aimed to describe the current obstetric anesthesia practices in the Sultanate of Oman by performing a questionnaire-based survey.

Methods

The Ministry of Health-Centre approved the survey for Studies and Research, Sultanate of Oman (MOH-CSR/25057). A Google Form with 25 questions (seven general questions and 18 specific questions) was initially shared in a WhatsApp group of members of the Oman Society of Anaesthesia and Critical Care (OSACC). Anesthesiologists who were not members were contacted directly and responses were recorded.

Results

Responses were accepted until midnight on December 31, 2023. The number of responses received was 66. Variations in practices like less compliance to Enhanced Recovery After Surgery (ERAS) pathways, use of oxytocin, and choice of intrathecal opioids were observed. Labor analgesia was not practiced by 30.3% of respondents. The majority of respondents did not follow international recommendations regarding the use of the uterotonic drug oxytocin.

Conclusion

A lot of heterogeneity in the practice of obstetric anesthesia in the Sultanate of Oman was observed. The limitations included the relatively low number of responses and many aspects that were missed in the survey. The findings of this survey will help in establishing a national task force for obstetric anesthesia, which will guide the members of the task force to develop practice guidelines based on international recommendations and the latest evidence.

## Introduction

Obstetric anesthesia as a subspecialty of anesthesia is extremely challenging. The scope of obstetric anesthesiologists comprises but is not limited to, increasing the safety of expectant mothers and newborns. An obstetric anesthesiologist is expected to be proficient in cardiovascular, pain, and critical care aspects relevant to patient care while practicing obstetric anesthesia. Maternal acid-base imbalances, postpartum pain, hemorrhage, hypertension, and non-obstetric medical emergencies are among the conditions that obstetric anesthesiologists manage. In addition to managing near-miss cases, obstetric anesthesiologists are also involved in the development and implementation of prognosis and preventive early warning systems in obstetrics [1,2]. The sub-specialty has become more challenging as parturients present with advanced age, multiple comorbidities, and high-risk pregnancies such as pre-eclampsia, eclampsia, multiple gestation, autoimmune diseases, maternal addiction disorders, obesity, depression, pregnancies after infertility treatment, multipara status, congenital defects in the fetus, and intrauterine growth retardation.

No published survey from the Middle East has investigated obstetric anesthesia practices in the region. The present survey aimed to describe the current obstetric anesthetic practices in the Sultanate of Oman by performing a questionnaire-based survey.

The results of this survey were presented as an abstract at the 18th World Congress of Anesthesiology (WCA2024), held in Singapore from March 3rd to 7th, 2024.

## Materials and methods

Oman, officially known as the Sultanate of Oman, is a West Asian country and one of the countries belonging to the Middle East. In the year 2021, the population of the Sultanate of Oman was 4.58 million. The same year, the crude birth rate was 22.88 per 1000 population. There are 91 hospitals (government and private) in the Sultanate of Oman out of which 79 hospitals provide maternity services (normal vaginal delivery and/or cesarean deliveries). Researchers in anesthesiology have employed survey research to examine clinical, educational, and professional areas. A questionnaire-based survey helps in understanding existing practices and adherence to recommended practices.

The primary aim of the survey was to gather data on obstetric anesthesia practices from obstetric anesthesia and labor analgesia providers in various hospitals across Oman. There was no previously conducted survey investigating obstetric anesthesia practices in Oman. Therefore, there were no previous responses available to prepare a questionnaire. A Google Form comprised 25 questions with either single-response answers or multiple-answers: 25 questions (seven were general and 18 were specific to perioperative practices). The survey primarily focused on pre-operative instructions, types of anesthesia, intraoperative practices including the use of oxytocin, Enhanced Recovery After Surgery (ERAS) pathways, intraoperative warming, nausea/vomiting prophylaxis, regional anesthesia (RA), multimodal analgesia, and labor analgesia. Seven questions of the survey were not analyzed. They are the name of the responder (optional), name of the hospital, location of work, designation, mobile number (optional), WhatsApp number (optional), and email ID (optional).

The following is the link to the Google form that was used: https://docs.google.com/forms/d/1SYtHLRQgPwu1uvqOQPzZxgNhDyoj_97YJ5-qg02LUmo/edit

The form was initially shared in a WhatsApp group of members of the Oman Society of Anaesthesia and Critical Care (OSACC). Anesthesiologists who were not members were contacted directly and responses were collected. The hospitals having functional obstetric anesthesia services were specifically targeted. The details of the responses were collected from the results section in the Google form. The responses were entered in a table as percentages and frequencies. The responses were collected till midnight of December 31, 2023, after which no more responses were accepted.

## Results

After we stopped accepting responses, we had a total of 66 responses. Table 1 shows the responses to all the 18 questions received from all 66 participants. The RA was the most commonly performed anesthesia technique (63 participants, 95.5% of responders) for lower segment cesarean section (LSCS). The counseling for the anesthesia plan was done by 65 responders (98.5%). The fasting guidelines were variable from six hours for solids and liquids by 21 (31.8%) responders, six hours for solids and four hours for liquids by 10 (15.2%) responders, and six hours for solids and two hours for liquids by 35 (53%) responders. Out of 66 responders, 54 (81.8%) used intrathecal opioids as an adjuvant. Intrathecal morphine as an adjuvant was used by three (4.5%) responders, 53 (80.3%) responders used fentanyl, and 10 (15.2%) responders did not use any adjuvants. The most common spinal needle used was Whitacre, by 41 (62.1%) responders followed by Quincke (20 or 30.3% responders). The cut-off platelet count for spinal anesthesia was 80,000 per cubic mm in 31 (47%) responders and 50,000 per cubic mm in 19 (28.8%) responders. For epidural placement, the platelet count cut-off was 100,000 per cubic mm in 28 (42.4%) responders, 80,000 per cubic mm in 20 (30.3%) responders, and 75,000 per cubic mm in 15 (22.7%) responders. Suxamethonium was the most common muscle relaxant used for general anesthesia (GA) by 44 (66.7 %) responders followed by rocuronium which was by nine (13.6%) responders. The use of the uterotonic agent oxytocin was very variable. Out of 66 responders, 54 (81.8%) used a 5 IU (international units) bolus followed by an infusion, nine (13.6%) used a 5 IU bolus with additional infusions as needed based on the obstetrician's request, and three (4.6%) started an infusion right away. The ERAS pathways were utilized by 43 (65.2%) responders and not utilized by 23 (34.8%) of responders. Labor analgesia was not practiced by 20 (30.3%) responders.

**Table 1:** Response to practice-specific questions LSCS: lower-segment cesarean section; ERAS: Enhanced Recovery After Surgery, cu.mm: cubic millimeter, LOR: loss of resistance; TENS: transcutaneous electrical nerve stimulation; IU: international units; USG: ultrasonography; IV: intravenous; TAP: transversus abdominis plane; QL: quadratus lumborum; GA: general anesthesia; NBM: nil by mouth; NSAIDs: non-steroidal anti-inflammatory drugs

Questions (n = 18)	Percentage (frequency) (total responses = 66)
Commonly practiced anesthesia technique for LSCS	
General anesthesia	4.5% (3)
Regional anesthesia	95.5% (63)
Are patients counseled preoperatively before elective LSCS regarding the type of anesthesia?	
Yes	98.5% (65)
No	1.5% (1)
Duration of NBM orders before elective LSCS	
6 hours (for solids, liquids)	31.8% (21)
6 hours for solids, 4 hours for liquids	15.2% (10)
6 hours for solids, 2 hours for liquids	53% (35)
Do you add intrathecal opioids for spinal anesthesia?	
Yes	81.8% (54)
No	18.2% (12)
If yes, which intrathecal opioid do you use?	
Morphine	4.5% (3)
Fentanyl	80.3% (53)
Not applicable	15.2% (10)
What multimodal analgesia is offered for LSCS under GA?	
Skin infiltration	65.2% (43)
NSAIDs	77.3% (51)
TAP block	43.9% (29)
QL block	4.5% (3)
IV/oral opioids	65.2% (43)
Acetaminophen	95.5% (63)
Patient-controlled analgesia	34.8% (23)
The needle of choice for spinal anesthesia	
Quincke	30.3% (20)
Whitacre	62.1% (41)
No preference	7.6% (5)
For general anesthesia, what is your choice of muscle relaxant?	
Suxamethonium	66.7% (44)
Rocuronium	13.6% (9)
Cisatracurium/Atracurium	19.7% (13)
The practice of use of uterotonic agent (oxytocin) in your place?	
5 IU bolus and infusion	81.8% (54)
5 IU bolus and as required	13.6% (9)
10 IU infusion	4.6% (3)
Do you implement ERAS pathways for LSCS?	
Yes	65.2% (43)
No	34.8% (23)
Do you give supplemental oxygen to patients who received spinal anesthesia?	
Yes	80.3% (53)
No	19.7% (13)
Do you use a patient warming system intraoperatively? (Bair Hugger, warming blankets)	
Yes	90.9% (60)
No	9.1% (6)
Do you use prophylactic anti-emetics for LSCS? (ondansetron, dexamethasone)	
Yes	77.3% (51)
No	22.7% (15)
What is your cut-off platelet count for spinal anesthesia?	
50,000/cu.mm	28.8% (19)
60,000/cu.mm	Zero (0)
75,000/cu.mm	24.2% (16)
80,000/cu.mm	47% (31)
What is your cut-off platelet count for epidural anesthesia/labor epidural?	
100,000/cu.mm	42.4% (28)
80,000/cu.mm	30.3% (20)
75,000/cu.mm	22.7% (15)
50,000/cu.mm	4.6% (3)
Use of USG for anticipated/encountered difficult spinal anesthesia	
No	80.3% (53)
Yes	19.7% (13)
What labor analgesia is practiced at your hospital?	
Epidural	62.1% (41)
Entonox	6.1% (4)
TENS	0% (0)
Others	1.5% (1)
We do not practice labor analgesia	30.3% (20)
For epidural space LOR, what is your preference?	
Air	72.7% (48)
Saline	27.3% (18)
Others (Epidrum, Episure)	None (0)

## Discussion

Summary of surveys from various countries

Many countries evaluated the practices of obstetric anesthesia using questionnaires. This is probably the first-ever survey from the Middle East. We found a lot of heterogeneity in the practice of obstetric anesthesia in the Sultanate of Oman. The practices could be different due to different countries of training and different seniority levels.

Staikou et al. evaluated the current practices in obstetric anesthesia using a 19-point questionnaire that was uploaded to the website of the European Society of Anaesthesia [3]. The European anesthesiologists preferred spinal anesthesia over GA, saving the latter for situations in which bleeding is likely to occur. The use of phenylephrine and fluid co-loading started to gain popularity at that time. On the other hand, despite substantial disagreement, the majority of anesthesiologists continued to advocate for cricoid pressure, standard supplemental oxygen, and high doses of oxytocin.

Staikou et al. reported that the majority of cesarean deliveries (CDs) in Greek public hospitals used RA, with a percentage of roughly 70% [4]. The most widely used method was the single-shot spinal block, particularly in hospitals outside of Athens. Very few hospitals, particularly those outside of Athens, use epidural labor analgesia, and anesthesiologists are not typically involved in labor analgesia or the peripartum care of women having a typical vaginal delivery. The questionnaire, however, did not look into the satisfaction levels of parturients regarding the anesthetic/analgesic techniques and services offered.

In a population-based study by Lai et al., which included 25,606 patients, spinal anesthesia was the most often used anesthetic method for CDs in Taiwan [5]. Preeclampsia, emergency LSCS, previous LSCS, early or threatened labor, and antepartum hemorrhage are important factors that influence GA in CS deliveries. The authors concluded that the use of GA had decreased gradually from 5.5% in 2000 to 3.9% in 2008, with spinal anesthesia being utilized more commonly in Taiwan during the past decade.

Juang et al. analyzed the patterns of obstetric anesthesia using National Anesthesiology Clinical Outcomes Registry data [6]. The data revealed that there were 218,285 cesarean sections done between 2010 and 2015. The GA was used in 5.8% of all CDs and 14.6% of emergent CDs. They found that there was a higher rate of GA for LSCS done in university hospitals, especially during after hours and on weekends, and also for LSCS in participants with American Society of Anesthesiologists class III or higher and 18 years of age or younger. Marcus et al. analyzed the practice of obstetric anesthesia in Germany by sending questionnaires to 709 departments [7]. Spinal anesthesia was the most commonly used method, accounting for 90.8% of the patients analyzed. Benhamou et al. surveyed France in 2005 to understand the anesthetic practices with scheduled LSCS with a 26-item questionnaire [8]. The analysis revealed that the anesthetic techniques used were single-shot spinal, epidural, combined spinal epidural, and GA in decreasing order (92.5, 4.5, 2, and 1%, respectively).

Weiniger et al. conducted a survey to understand the organization and the practice of obstetric anesthesia in Israel by sending a questionnaire to 11 hospitals providing anesthesia for obstetric patients [9]. The use of GA was 15% (0.5-50), epidural 14.5% (0-99.5), spinal 68% (0-98), or combined spinal-epidural technique 0% (0-30) for CD (elective and emergency combined). In 50% (4-93) of deliveries, labor analgesia was administered via epidural procedures; in 0.5% (0-90) of deliveries, nitrous oxide was used. For labor analgesia (17/25), postpartum hemorrhage (12/25), aspiration prophylaxis (15/25), and maternal resuscitation (8/25), written protocols were available. The authors concluded that labor epidural analgesia was used sufficiently, but that there were significant staffing disparities, a dearth of written protocols, and a shortage of obstetric anesthesia specialists.

To evaluate the existing obstetric anesthesia practices in Austria, Oji-Zurmeyer et al. emailed questionnaires to key anesthesiologists from obstetric anesthesia departments of 81 hospitals registered at the Austrian Ministry of Health [10]. For elective LSCS, spinal anesthesia was the primary anesthetic technique offered by all responders. Of the respondents, three (5%) used long-acting intrathecal morphine, and 18 (28%) did not regularly use intrathecal opioids. Acute postoperative pain control through wound infiltration was implemented in two (3%) of the respondent units. In 14 (22%) departments, a transversus abdominis plane block was provided as a rescue analgesic. In two (3%) of the responding hospitals, prophylactic phenylephrine infusion was used to treat hypotension resulting from spinal anesthesia. Thirty-one (48%) of the respondents routinely administered prophylactic antibiotics before skin incision.

Obstetric anesthesia workforce surveys were conducted in the United States in 1981, 1992, and 2001, and a 10-year update was done in 2012 [11-14]. Traynor et al. surveyed to update the existing practices after 30 years, in 2016. The hospitals were graded in various strata as follows: I ≥ 1500 annual births (n = 341), II ≥ 500 to 1499 annual births (n = 438), and III < 500 annual births (n = 414). The in charge of obstetric services was emailed the survey questionnaire to get responses. Labor epidural analgesia was available 24 hours per day in all strata I hospitals. There were high rates of in-house coverage (86.3%) of anesthesiology services in stratum I hospitals for obstetrics. The use of patient-controlled epidural analgesia in stratum I hospitals was reported to be 35% in 2001 and 77.6%. Certified Registered Nurse Anesthetists were reported to provide obstetric anesthesia services in 68% of stratum III hospitals.

Summary of practices in the Sultanate of Oman

A few responses from the survey need to be mentioned. Intrathecal morphine as an adjuvant was used by three (4.5%) responders, 53 (80.3%) responders used fentanyl, and 10 (15.2%) responders did not add any adjuvant to intrathecal bupivacaine. Intrathecal morphine is considered the gold standard single-shot drug for postoperative pain with the duration of action of intrathecal morphine between 14 and 36 hours. The safety and efficacy also depend on the dose used. A dose of 50-150 mcg is considered efficacious with minimal adverse events like pruritus and respiratory depression [15-17].

The principles of ERAS pathways are increasingly being adapted and studied in the context of obstetric anesthesia to address the unique challenges and considerations of the pregnant population undergoing LSCS [18]. The ERAS gained so much popularity that obstetric anesthesiologists coined a new terminology called Enhanced Recovery for Obstetric Surgery (EROS) [19]. While ERAS and EROS place a strong emphasis on RA, the anesthesiologist should have the final say over the anesthetic plan based on the patient's characteristics, the current circumstances, and the LSCS category. The anesthesiologist should offer all ERAS pathways, including multimodal, opioid-sparing anesthesia, even if GA is considered due to specific circumstances. The Sultanate of Oman has a policy document on ERAS in obstetrics. However, only 43 (65.2%) responders used ERAS pathways in obstetrics. For women delivering by cesarean section, oxytocin (5 IU by slow intravenous injection) should be used to encourage contraction of the uterus and to decrease blood loss (Grade of recommendation: A) [20]. However, the survey revealed that 5 IU and infusion were used by 54 (81.8%) respondents, three (4.6%) respondents used 10 IU as an infusion, and only nine (13.6%) respondents replied administering 5 IU as a bolus and thereafter as necessary. Existing evidence mentions that the results do not differ between air and saline in terms of the loss of resistance technique for identification of the epidural space and reduction of complications. Most of the studies involved parturients [21]. Regarding the use of the type of spinal needles, 41 (62.1%) respondents preferred Whitacre, 20 (30.3%) respondents preferred Quincke, and five (7.6%) respondents had no preference. The survey did not enquire about the size of the spinal needle as 25-G is the lowest size available in Oman. The results of a systematic review and network meta-analysis recommend using a 26-G atraumatic needle to enable successful insertion while avoiding post-dural puncture headaches. However, in case of non-availability, clinicians may select the best available options [22].

Both suxamethonium and rocuronium are considered safe medications for rapid-sequence induction for LSCS. The choice of muscle relaxant is usually associated with familiarity and circumstances. Literature has described Scoline (suxamethonium) induced apnea as a result of inherited or acquired deficiency of the cholinesterase enzyme causing prolonged muscle relaxation after a single dose [23,24]. Patients with a previous history of delayed recovery after GA after ruling out metabolic factors like hypothyroidism should arouse suspicion of Scoline apnea. In such cases, rocuronium should be used. Sugammadex is a modified gamma-cyclodextrin that can completely reverse rocuronium. Obstetric Anaesthetists' Association and Difficult Airway Society recommends the use of sugammadex for antagonizing neuromuscular blockade achieved with rocuronium if it is available [25].

Limitations

There were a lot of limitations to this survey. The limitation was a small sample of the respondents. Many aspects that could have improved the overall assessment of the obstetric practices were missed in the survey. The response rate could not be determined. A few more questions that were missed in the questionnaire were routine use of aspiration prophylaxis (for both elective and emergency LSCS), use of gastric ultrasound for assessing the gastric residual volume, the use of indwelling epidural catheter cited for labor analgesia for LSCS, and the use of a supraglottic airway device especially in an elective and adequately fasting patient for LSCS. We also did not enquire if all hospitals have policies and protocols related to obstetric anesthesia labor analgesia, transfusion trigger, massive obstetric hemorrhage, ERAS pathways, thromboprophylaxis, and criteria for ICU/high-dependency unit admission. We also did not enquire about the availability of sugammadex and its routine use for antagonizing rocuronium-induced neuromuscular blockade. Furthermore, we did not enquire if a particular hospital audits patient satisfaction scores from parturients.

## Conclusions

At present, there is a lot of heterogeneity in the practice of obstetric anesthesia in the Sultanate of Oman. The findings of this survey will help in establishing a national task force for obstetric anesthesia, which will guide the members of the task force to develop practice guidelines based on international recommendations and the latest evidence. Once established, compliance in implementing these practice guidelines will be assessed.
